# Intranasal exposure of African green monkeys to SARS-CoV-2 results in acute phase pneumonia with shedding and lung injury still present in the early convalescence phase

**DOI:** 10.1186/s12985-020-01396-w

**Published:** 2020-08-18

**Authors:** Robert W. Cross, Krystle N. Agans, Abhishek N. Prasad, Viktoriya Borisevich, Courtney Woolsey, Daniel J. Deer, Natalie S. Dobias, Joan B. Geisbert, Karla A. Fenton, Thomas W. Geisbert

**Affiliations:** 1 Department of Microbiology and Immunology, University of Texas Medical Branch, Galveston, TX 77555 USA; 2 Galveston National Laboratory, University of Texas Medical Branch, Galveston, TX 77555 USA

**Keywords:** Coronavirus, SARS-CoV-2, COVID-19, Nonhuman primate, Animal models

## Abstract

We recently reported the development of the first African green monkey (AGM) model for COVID-19 based on a combined liquid intranasal (i.n.) and intratracheal (i.t.) exposure to severe acute respiratory syndrome coronavirus 2 (SARS-CoV-2). Here, we followed up on this work by assessing an i.n. particle only route of exposure using the LMA mucosal atomization device (MAD). Six AGMs were infected with SARS-CoV-2; three animals were euthanized near the peak stage of virus replication (day 5) and three animals were euthanized during the early convalescence period (day 34). All six AGMs supported robust SARS-CoV-2 replication and developed respiratory disease. Evidence of coagulation dysfunction as noted by a transient increases in aPTT and circulating levels of fibrinogen was observed in all AGMs. The level of SARS-CoV-2 replication and lung pathology was not quite as pronounced as previously reported with AGMs exposed by the combined i.n. and i.t. routes; however, SARS-CoV-2 RNA was detected in nasal swabs of some animals as late as day 15 and rectal swabs as late as day 28 after virus challenge. Of particular importance to this study, all three AGMs that were followed until the early convalescence stage of COVID-19 showed substantial lung pathology at necropsy as evidenced by multifocal chronic interstitial pneumonia and increased collagen deposition in alveolar walls despite the absence of detectable SARS-CoV-2 in any of the lungs of these animals. These findings are consistent with human COVID-19 further demonstrating that the AGM faithfully reproduces the human condition.

## Introduction

The unprecedented pandemic of COVID-19 caused by severe acute respiratory syndrome coronavirus 2 (SARS-CoV-2) has had devastating effects on public health and the global economy. Considerable resources have been allocated by governments, philanthropic organizations, and private companies in an attempt to expedite the development of vaccines and treatments to combat COVID-19. With the rapid development of 24 preventative vaccines in clinical evaluation [1], and nearly 200 more in the pipeline [2], coupled with the availability of nearly 300 candidate antivirals and disease modulators [2] it is impossible to investigate the safety and efficacy of all of these various interventions in humans. Both small animal models and nonhuman primates (NHP) may prove valuable in triaging the most promising medical countermeasures prior to use in humans. Hamsters and ferrets are currently being used as immunocompetent small animal models of COVID-19 [3–5] while several NHP models have been quickly developed [6–12]. Among the nonhuman primate models evaluated the African green monkey (AGM) appears to best recapitulate the most salient features of human COVID-19 [10–12].

We recently reported the development of the first AGM model for COVID-19 and showed that back-challenge of animals with SARS-CoV-2 5 weeks after initial exposure resulted in protection from reinfection [10]. In this study the AGMs were exposed to SARS-CoV-2 by a combination of the intranasal (i.n.) and intratracheal (i.t.) routes with the virus delivered in liquid media. As a natural extension of this initial work we sought to assess the pathogenesis of SARS-CoV-2 in AGMs exposed by the i.n. route only using the LMA Mucosal Atomization Device (MAD). Previous studies with another respiratory virus, Nipah virus, showed that there were no major differences in disease pathogenesis when virus was delivered to AGMs by a combined liquid-based i.n. and i.t. delivery [13] or by the LMA MAD system [14]. The LMA MAD was developed for the efficient and safe delivery of test particles and is currently employed to administer US FDA approved drugs for i.n. delivery. The LMA MAD delivers atomized particles that range in size from 30 to 100 μm, which is highly consistent with the size of droplets exhaled by humans due to coughing [15]. In addition, in our previous work as the AGMs were back challenged with SARS-CoV-2 it was impossible to assess tissue pathology during convalescence after primary challenge. Here, we focused on assessing the pathogenesis of SARS-CoV-2 infection in AGMs when administered as 30 to 100 μm particles and on evaluating virus shedding and lung pathology during early convalescence.

## Materials and methods

### Virus

The virus (SARS-CoV-2/INMI1-Isolate/2020/Italy) was isolated on January 30, 2020 from the sputum of the first clinical case in Italy, a tourist visiting from the Hubei province of China that developed respiratory illness while traveling [16]. The virus was initially passaged twice (P2) on Vero E6 cells; the supernatant and cell lysate were collected and clarified following a freeze/thaw cycle. This isolate is certified mycoplasma and Foot-and-Mouth Disease virus free. The complete sequence was submitted to GenBank (MT066156) and is available on the GISAID website (BetaCoV/Italy/INMI1-isl/2020: EPI_ISL_410545) upon registration. For in vivo challenge, the P2 virus was propagated on Vero E6 cells and the supernatant was collected and clarified by centrifugation making the virus used in this study a P3 stock.

### Animal challenge

SARS-CoV-2 seronegative AGMs (*Chlorocebus aethiops*) (6 females) (St Kitts origin, Worldwide Primates, Inc.) were randomized into two cohorts where one group (*n* = 3) was scheduled for euthanasia at 5 dpi and the other at 34 dpi. Animals were anesthetized with ketamine and inoculated with a target dose of 3.0 × 10^6^ PFU of SARS-CoV-2 (SARS-CoV-2/INMI1-Isolate/2020/Italy) using the LMA MAD, with the dose being equally divided between each nostril. All animals were longitudinally monitored for clinical signs of illness including temperature (measured by surgically implanted DST micro-T small implantable thermo loggers (Star-Oddi, Gardabaer, Iceland)), respiration quality, and clinical pathology. All measurements requiring physical manipulation of the animals were performed under sedation by ketamine. Mucosal swabs were obtained using sterile swabs inserted into the mucosal cavity, gently rotated to maximize contact with the mucosal surface, and deposited into 2.0 mL screw-top tubes containing sterile MEM media supplemented to 2% with FBS.

### RNA isolation from SARS-CoV-2-infected AGMs

On specified procedure days (days 0, 2, 3, 4, 5, 7, 12, 15, 21, 28, 34), 100 μl of blood was added to 600 μl of AVL viral lysis buffer (Qiagen) for virus inactivation and RNA extraction. Following removal from the high containment laboratory, RNA was isolated from blood and swabs using the QIAamp viral RNA kit (Qiagen).

### Detection of SARS-CoV-2 load

RNA was isolated from blood and mucosal swabs and assessed using the CDC SARS-CoV-2 N2 assay primers/probe for reverse transcriptase quantitative PCR (RT-qPCR) [17]. SARS-CoV-2 RNA was detected using One-step probe RT-qPCR kits (Qiagen) run on the CFX96 detection system (Bio-Rad), with the following cycle conditions: 50 °C for 10 min, 95 °C for 10 s, and 45 cycles of 95 °C for 10 s and 55 °C for 30 s. Threshold cycle (*C*_*T*_) values representing SARS-CoV-2 genomes were analyzed with CFX Manager Software, and data are presented as GEq. To generate the GEq standard curve, RNA was extracted from supernatant derived from Vero E6 cells infected with SARS-CoV-2/INMI1-Isolate/2020/Italy was extracted and the number of genomes was calculated using Avogadro’s number and the molecular weight of the SARS-CoV-2 genome.

Infectious virus was quantitated by plaque assay on Vero E6 cells (ATCC CRL-1586) from all blood plasma and mucosal swabs, and bronchoalveolar lavage (BAL) samples. Briefly, increasing 10-fold dilutions of the samples were adsorbed to Vero E6 cell monolayers in duplicate wells (200 μl). Cells were overlaid with EMEM medium plus 1.25% Avicel, incubated for 2 days, and plaques were counted after staining with 1% crystal violet in formalin. The limit of detection for this assay is 25 PFU/ml.

### Hematology and serum biochemistry

Total white blood cell counts, white blood cell differentials, red blood cell counts, platelet counts, hematocrit values, total hemoglobin concentrations, mean cell volumes, mean corpuscular volumes, and mean corpuscular hemoglobin concentrations were analyzed from blood collected in tubes containing EDTA using a Vetscan HM5 hematologic analyzer (Abaxis). Serum samples were tested for concentrations of albumin, amylase, alanine aminotransferase (ALT), aspartate aminotransferase (AST), alkaline phosphatase (ALP), blood urea nitrogen (BUN), calcium, creatinine (CRE), C-reactive protein (CRP), gamma-glutamyltransferase (GGT), glucose, total protein, and uric acid by using a Piccolo point-of-care analyzer and Biochemistry Panel Plus analyzer discs (Abaxis). Partial pressures of CO_2_ and O_2_ were obtained using an iSTAT Alinity hematological analyzer (Abbott).

### Serum neutralization assay

Neutralization titers were calculated by determining the dilution of serum that reduced 50% of plaques (PRNT_50_). A standard 100 PFU amount of SARS-CoV-2 was incubated with two-fold serial dilutions of serum samples for 1 hour. The virus-serum mixture was then used to inoculate Vero E6 cells for 60 min. Cells were overlaid with EMEM medium plus 1.25% Avicel, incubated for 2 days, and plaques were counted after staining with 1% crystal violet in formalin.

### ELISA

SARS-CoV-2-specific IgG antibodies to nucleoprotein were measured in sera by ELISA at the indicated time points. Nucleoprotein ELISA kits were kindly provided by Zalgen Labs, LLC. Sera were initially diluted 1:100 and then two-fold through 1:25,600 in 4 in (1 x PBS with 0.02% Tween-20). After a one-hour incubation, plates were washed six times with wash buffer (1 x PBS with 0.2% Tween-20) and incubated for an hour with a 1:5000 dilution of horseradish peroxidase conjugated anti-primate IgG antibody (Fitzgerald Industries International; Cat: 43R-IG020HRP). Tetramethylbenzidine was used to develop the reaction; the reaction was stopped with methane-sulfonic acid and plates were read at a wavelength of 450 nm. Absorbance values were normalized by blank-subtracting values from wells incubated with sera from a SARS-CoV-2-naïve animal at the corresponding serum dilution. End-point titers were defined as the reciprocal of the last adjusted serum dilution with a value ≥0.20.

### Histopathology and immunohistochemistry

Necropsy was performed on all subjects euthanized at 5 dpi and 34 dpi. Tissue samples of all major organs were collected for histopathologic and immunohistochemical (IHC) examination and were immersion-fixed in 10% neutral buffered formalin for > 7 days. Specimens were processed and embedded in paraffin and sectioned at 5 μm thickness. For IHC, specific anti-SARS immunoreactivity was detected using an anti-SARS nucleocapsid protein rabbit primary antibody at a 1:800 dilution for 60 min (Novusbio). The tissue sections were processed for IHC using the ThermoFisher Scientific Lab Vision Autostainer 360 (ThermoFisher Scientific). Secondary antibody used was biotinylated goat anti-rabbit IgG (Vector Laboratories) at 1:200 for 30 min followed by Vector Streptavidin Alkaline Phosphatase at a dilution of 1:200 for 20 min (Vector Laboratories). Slides were developed with Bio-Red (Biopath) for 7 min and counterstained with hematoxylin for 1 minute. For IHC, specific anti-fibrin was detected using an anti-fibrin monoclonal mouse primary antibody at a 1:3200 dilution for 60 min (Sekisui Diagnostics). The tissue sections were processed for IHC using the ThermoFisher Scientific Lab Vision Autostainer 360 (ThermoFisher Scientific). Secondary antibody used was biotinylated goat anti-mouse IgG (Vector Laboratories) at 1:200 for 30 min followed by Vector Streptavidin Alkaline Phosphatase at a dilution of 1:200 for 20 min (Vector Laboratories). Slides were developed with Bio-Red (Biopath Laboratories) for 7 min and counterstained with hematoxylin for 1 minute. Tissues were stained following package instructions for collagen with the Trichrome One-Step Blue & Red Stain Kit (American MasterTech Scientific Laboratory Supplies).

## Results

### SARS-CoV-2 experimental infection of African green monkeys using the LMA MAD

We challenged six healthy, adult AGMs with a target dose of 3.0 × 10^6^ PFU of SARS-CoV-2 (SARS-CoV-2/INMI1-Isolate/2020/Italy) via intranasal inoculation with the LMA MAD (actual delivered dose of 2.8 × 10^6^ PFU). Three animals were euthanized at 5 days post-infection (dpi) which is thought to be the approximate time point of peak disease in AGMs [10], while the remaining three animals were euthanized at 34 dpi during early convalescence. Blood and mucosal swabs were sampled from all animals on days 0, 2, 3, 4, 5, and additionally on days 7, 9, 12, 15, 21, 28, and 34 for AGM-4, AGM-5, and AGM-6. BAL fluid collection was performed on days − 8, 3, and 5 for all animals, as well as 7 dpi for AGM-4, AGM-5 and AGM-6. Consistent with our previous report describing the development of the combined intranasal and intratracheal SARS-CoV-2 challenge model in AGMs [10], we did not observe overt signs of clinical illness in any AGMs in this study, other than decreased appetite or brief (single day) anorexia (Supp Table 1). Temperature was longitudinally monitored in 15 min increments for the entire study duration using surgically implanted temperature loggers; several animals (AGM-4, AGM-6) experienced brief (< 2 h) periods of mildly elevated temperatures at 3 dpi, and two animals (AGM-2, AGM-3) exhibited an abnormal temperature cycling pattern at 3 dpi (Supp Fig. 1).

**Fig. 1:**
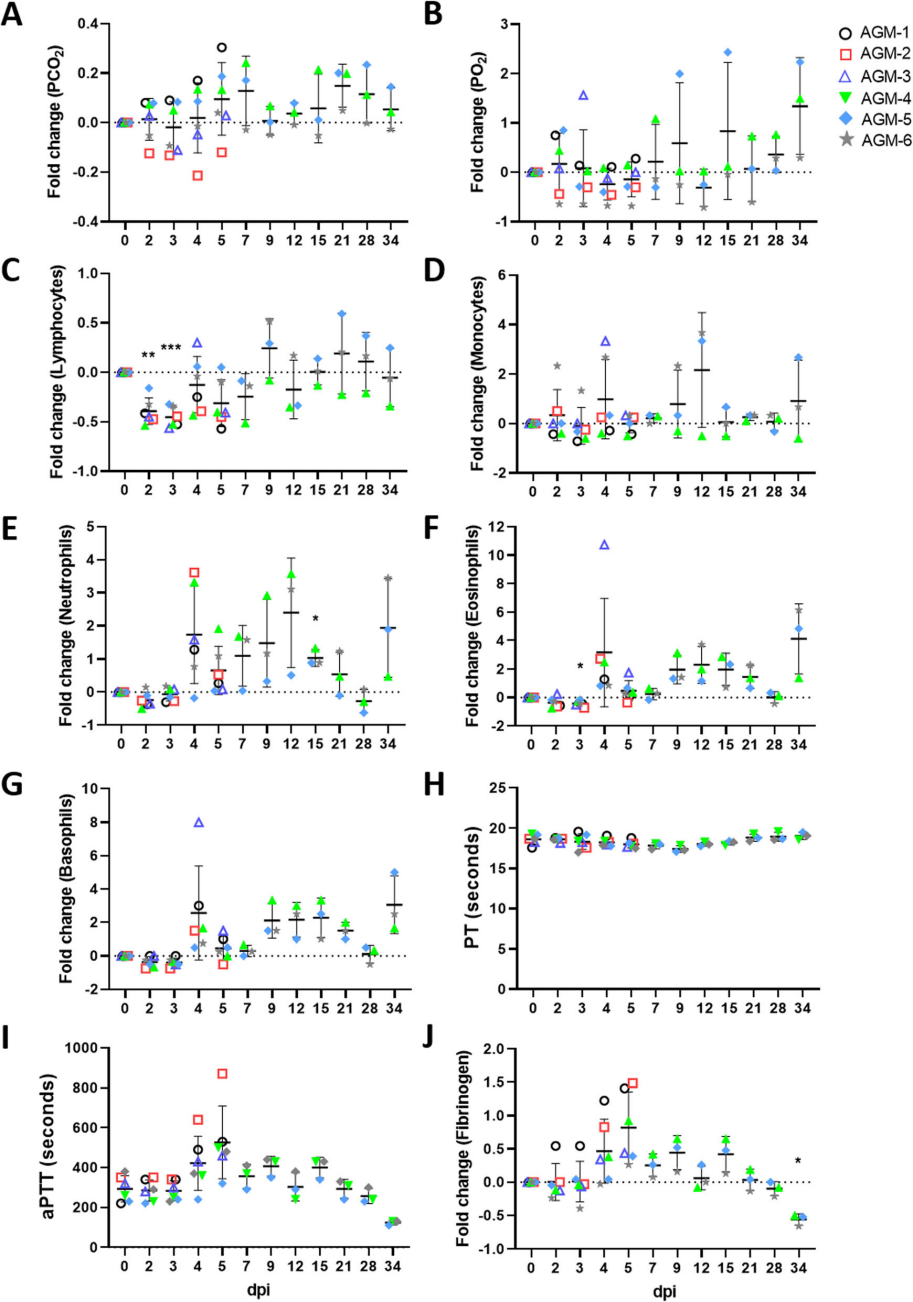
Hematological features of SARS-CoV-2 infection in AGMs. Blood gas (**a**, **b**), selected leukocyte populations (**c-g**), and coagulation assays (**h-j**) are shown. For parameters where fold change is used, fold change was determined by baseline (0 dpi) subtraction of each time point for each animal. Statistical significance was determined in Graphpad Prism 8.4.3 by mixed-effects analysis with the Geisser-Greenhouse correction without the assumption of sphericity, with multiple comparisons made using Dunnett’s post-hoc test and all comparisons made to baseline values (0 dpi). Asterisks denote significance: * = *p* ≤ 0.05, ** = *p* ≤ 0.01, *** = *p* ≤ 0.001. Two-tailed *p*-values were computed for all comparisons

As in our previous report, transient shifts in leukocyte populations, predominately manifested as lymphocytopenia (5/6 animals), thrombocytopenia (3/6 animals), and granulocytosis (defined by neutrophilia, eosinophilia, and/or basophilia) (6/6 animals) were observed, while markers for renal (BUN, CRE) and hepatic function (ALT, AST, ALP, GGT) remained unchanged for the most part, with the exception of mild (≤ 2-fold) increases in ALT (2/6 animals), and mild to moderate (1 to 16-fold) increases in CRP, a marker of acute systemic inflammation (5/6 animals) (Supp Table 1), although statistical significance was not reached for most parameters at most time points (Fig. 1). In addition, hypercapnia (defined here as ≥4 mmHg increase in dissolved CO_2_) was observed in 3/6 animals (Supp Table 1), which as we observed previously [10], appeared to follow a biphasic pattern (Fig. 1a, data shown as fold-change from baseline]).

All animals exhibited normal prothrombin times (PT) as compared to their individual baseline values; however, mild to moderate prolongation of the activated partial thromboplastin time (aPTT) was also observed in all animals through the acute phase of disease, most prominently in AGM-1 and AGM-2, indicating possible disorder of the intrinsic coagulation pathway (Fig. 1h, i); this was mirrored by increased levels of circulating fibrinogen (Fig. 1j). We previously showed that the pathways connected to IL-6 production are activated during SARS-CoV-2 infection of AGMs [10], indicating possible mechanisms of coagulopathy in the current study.

**Fig. 2:**
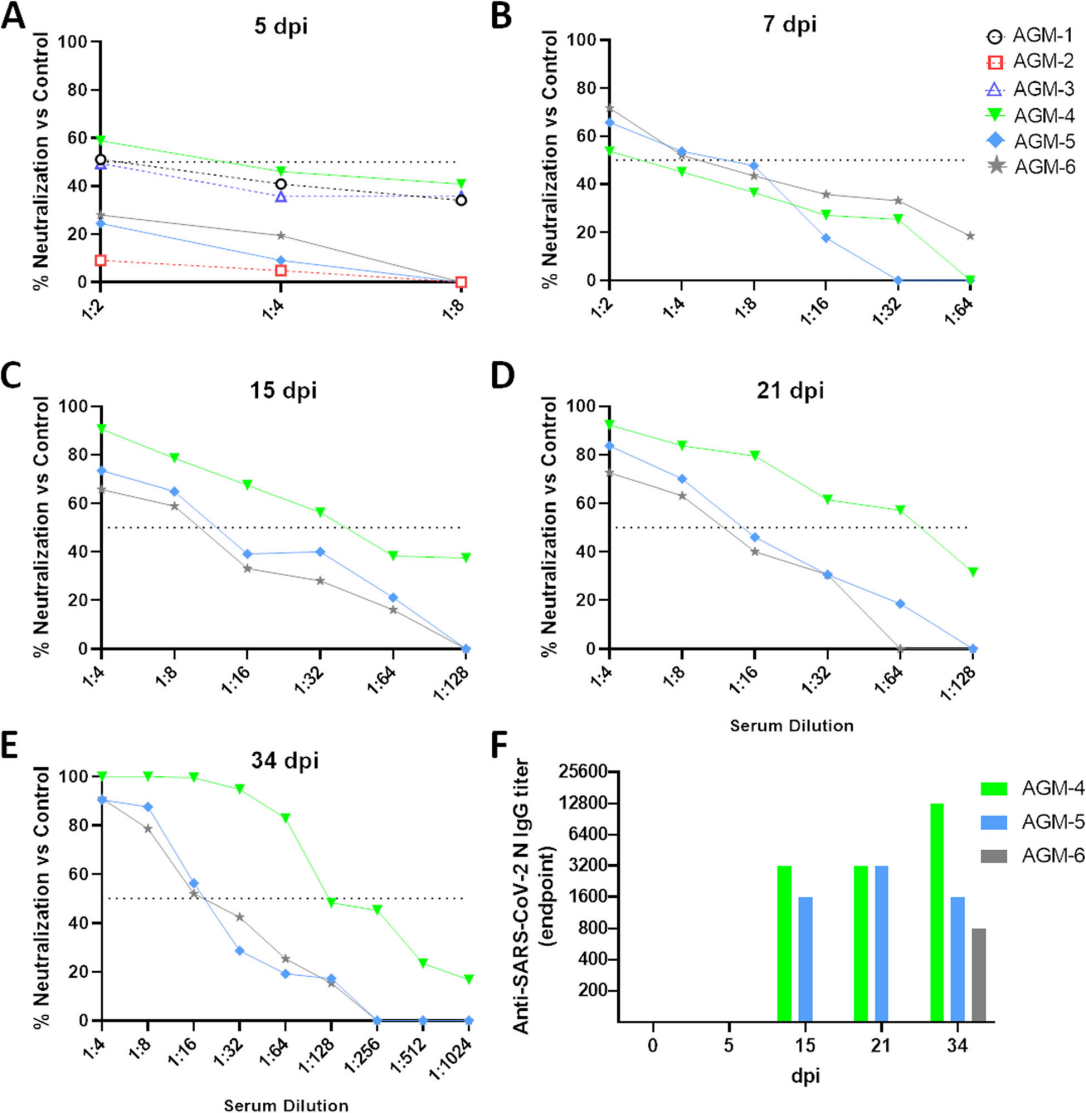
Serum neutralization and binding antibody titers in SARS-CoV-2 infected AGMs. Total anti-SARS-CoV-2 serum neutralization activity was determined for each animal by PRNT_50_ at the indicated time points (**a-e**). Anti-SARS-CoV-2 N protein specific IgG endpoint titers were quantified for each animal and time point by ELISA (**f**)

All animals seroconverted, with weakly neutralizing titers (as quantified by PRNT_50_) being detected as early as 5 dpi and gradually increasing in potency by 34 dpi, with terminal neutralizing antibody titers ranging from ~ 1:16–1:128 (Fig. 2a-e). We next quantified SARS-CoV-2 nucleoprotein specific IgG by ELISA (Fig. 2f). Seroconversion was not detected until day 15 in two animals (AGM-4 & AGM-5). Interestingly, not until 34 dpi was a modest level (1:800) of seroconversion detected in the third animal.

### Quantification of viral load in blood, mucosal swabs, and lungs

**Fig. 3:**
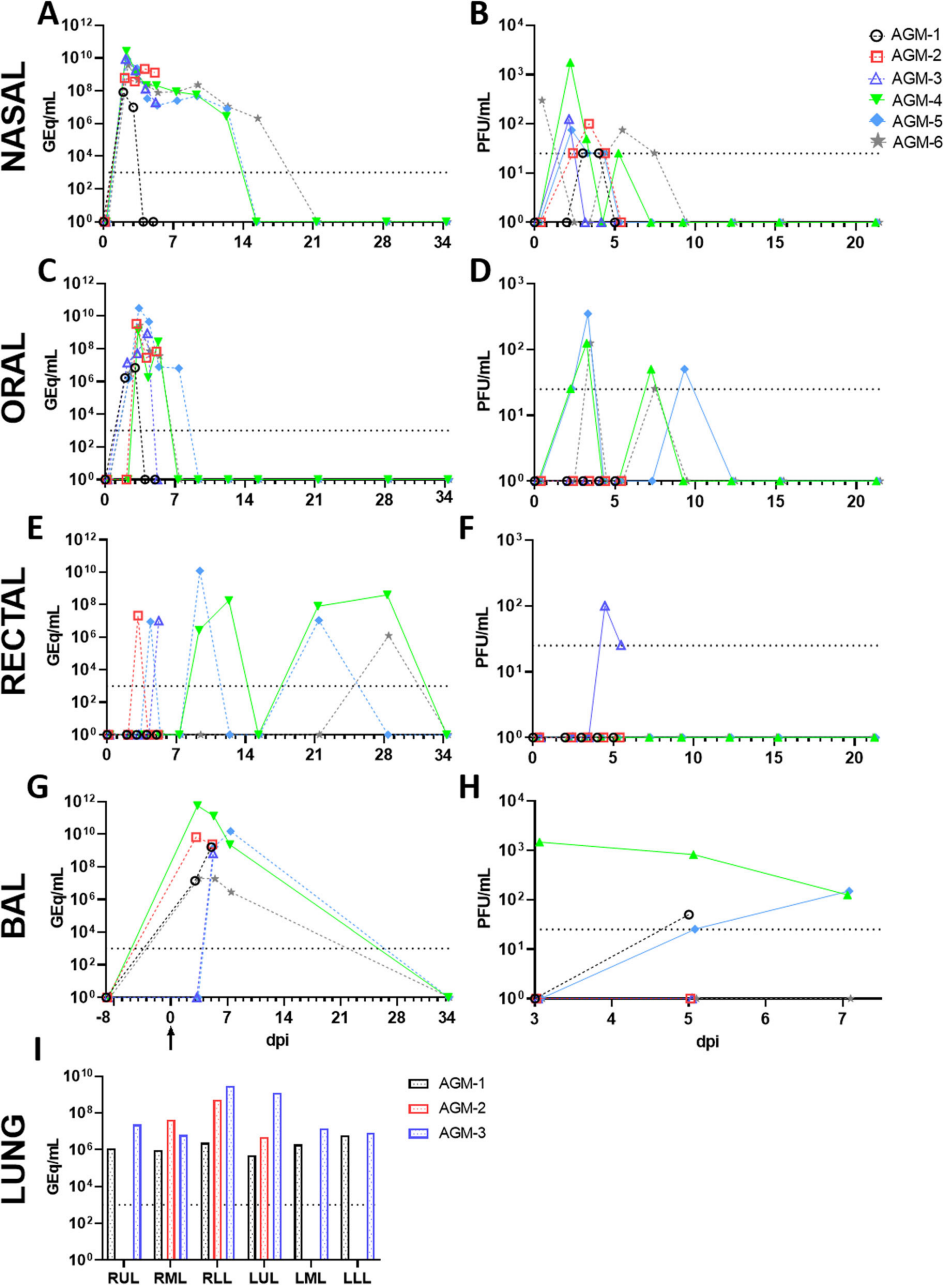
Quantification of SARS-CoV-2 infectious virus and vRNA in mucosal swabs, BAL fluid, and lung tissue. Viral load was quantified by detection of SARS-CoV-2 vRNA by RT-qPCR (**a**, **c**, **e**, **g**, **i**) or plaque titration (**b**, **d**, **f**, **h**). The limit of detection for each assay is indicated by horizontal dashed line (1000 GEq/mL for RT-qPCR, 25 PFU/mL for plaque titration). For both assays, data shown is the mean of two technical replicates of the same biological sample. Arrow in (**g**) indicates day of challenge. For panel I, RUL: right upper lung; RML: right middle lung; RLL: right lower lung; LUL: left upper lung; LML: left middle lung; LLL: left lower lung

Viral RNA (vRNA) was purified from whole blood, oral, nasal and rectal mucosa, and BAL fluid from all collection days, as well as from lung tissue harvested at necropsy. As we previously reported [10], we were unable to detect SARS-CoV-2 vRNA in whole blood by RT-qPCR, nor were we able to recover infectious virus in the plasma fraction by plaque assay, confirming a lack of either cell-associated or freely-circulating virus in the peripheral blood. SARS-CoV-2 vRNA and infectious virus was detected in the nasal mucosa from all animals as early as 2 dpi, with vRNA persisting in a single animal up to 15 dpi (Fig. 3a, b). Likewise, vRNA was detected in oral swabs from all animals beginning 2–3 dpi before falling below the limit of detection by 7 dpi, while low quantities of infectious virus (1–2 log_10_ PFU/mL) were only isolated from three animals (AGM-4, AGM-5, and AGM-6) (Fig. 3c, d). Remarkably, vRNA was transiently shed from the lower gastrointestinal tract up to 28 dpi (AGM-4 and AGM-6), although infectious virus could only be recovered from the rectal swab of a single animal (AGM-3) 4–5 dpi (Fig. 3e, f). vRNA was detected in BAL fluid from 4/6 animals 3 dpi and up to 7 dpi in all three animals held past 5 dpi, while infectious virus was recovered from 3/6 animals (Fig. 3g, h). Detectable quantities of vRNA were absent from lungs harvested during necropsy of AGMs euthanized 34 dpi, while 6–9 log_10_ GEq/g were detected from all three animals euthanized at 5 dpi (Fig. 3i).

### Gross pathology, histopathology, and immunohistochemistry

**Fig. 4:**
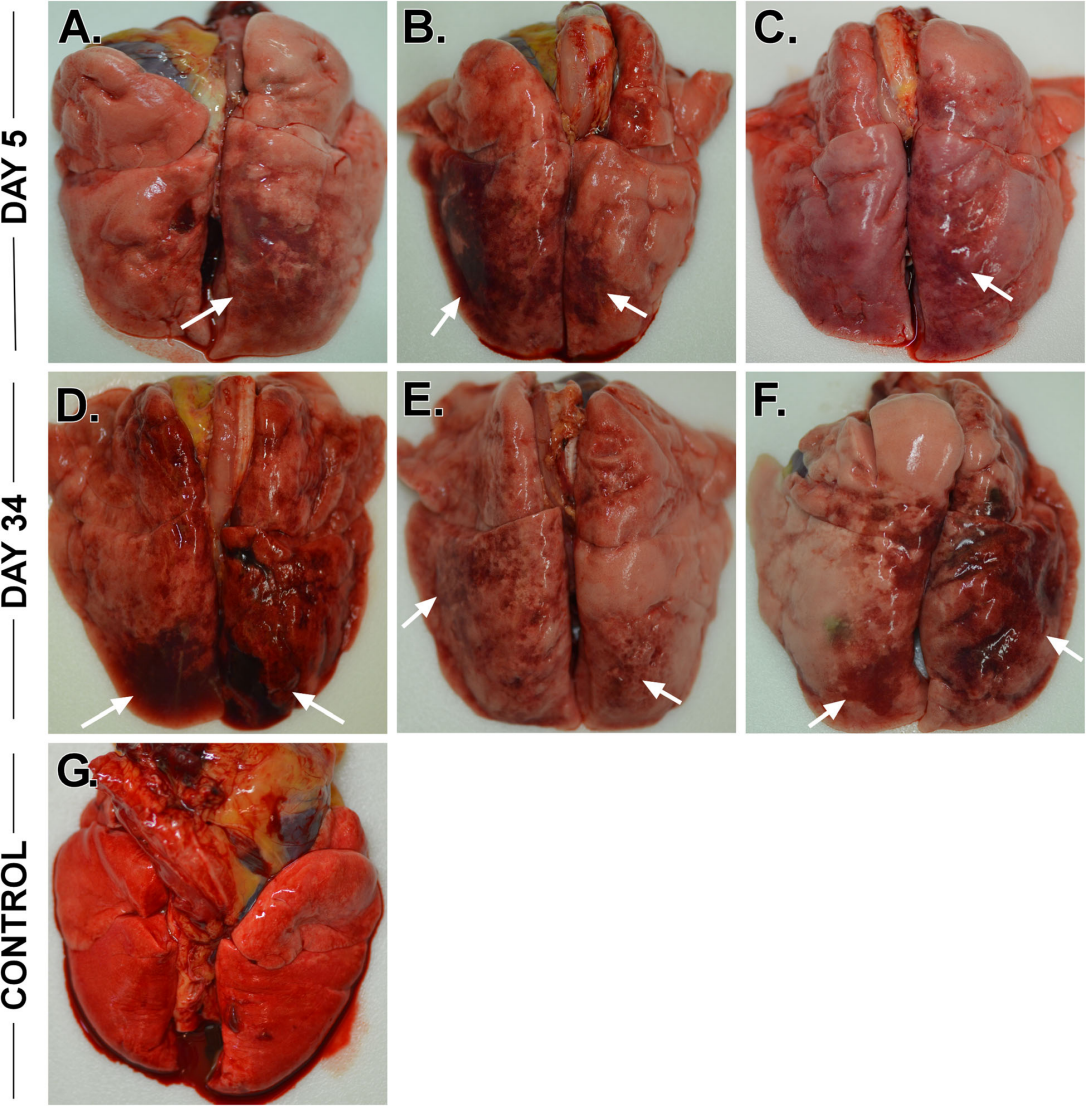
Gross lung pathology in AGMs infected with SARS-CoV-2. Dorsal view of lungs from AGM-1 (**a**), AGM-2 (**b**) and AGM-3(**c**) euthanized at 5 dpi with SARS-CoV-2 exhibiting mild to moderate locally extensive pulmonary consolidation with hyperemia and hemorrhage. Dorsal view of lungs from AGM-4 (**d**), AGM-5 (**e**) and AGM-6 (**f**) euthanized at 34 dpi with SARS-CoV-2 exhibiting mild to marked locally extensive pulmonary consolidation with hyperemia and hemorrhage. Dorsal view of control lungs with no significant lesions from SARS-CoV-2 negative AGM (**g**)

Necropsy was performed on all animals following euthanasia, and lungs were collected for gross examination and histopathological analysis. Consistent with our previous study utilizing a combined i.n. and i.t. inoculation route [10], all AGMs displayed varying degrees of pulmonary consolidation with hyperemia and hemorrhage, characterized by depressed and patchy dark red to light pink regions (Fig. 4**, arrows**). In all AGMs, the most severe lesions were located in the dorsal aspects of the lower lung lobes. A board-certified veterinary pathologist approximated lesion severity for each lung lobe (Supp Table 2). All AGMs at 5 dpi also had segmentally flaccid and gas distention of small intestines. There were no other significant gross lesions.

**Fig. 5:**
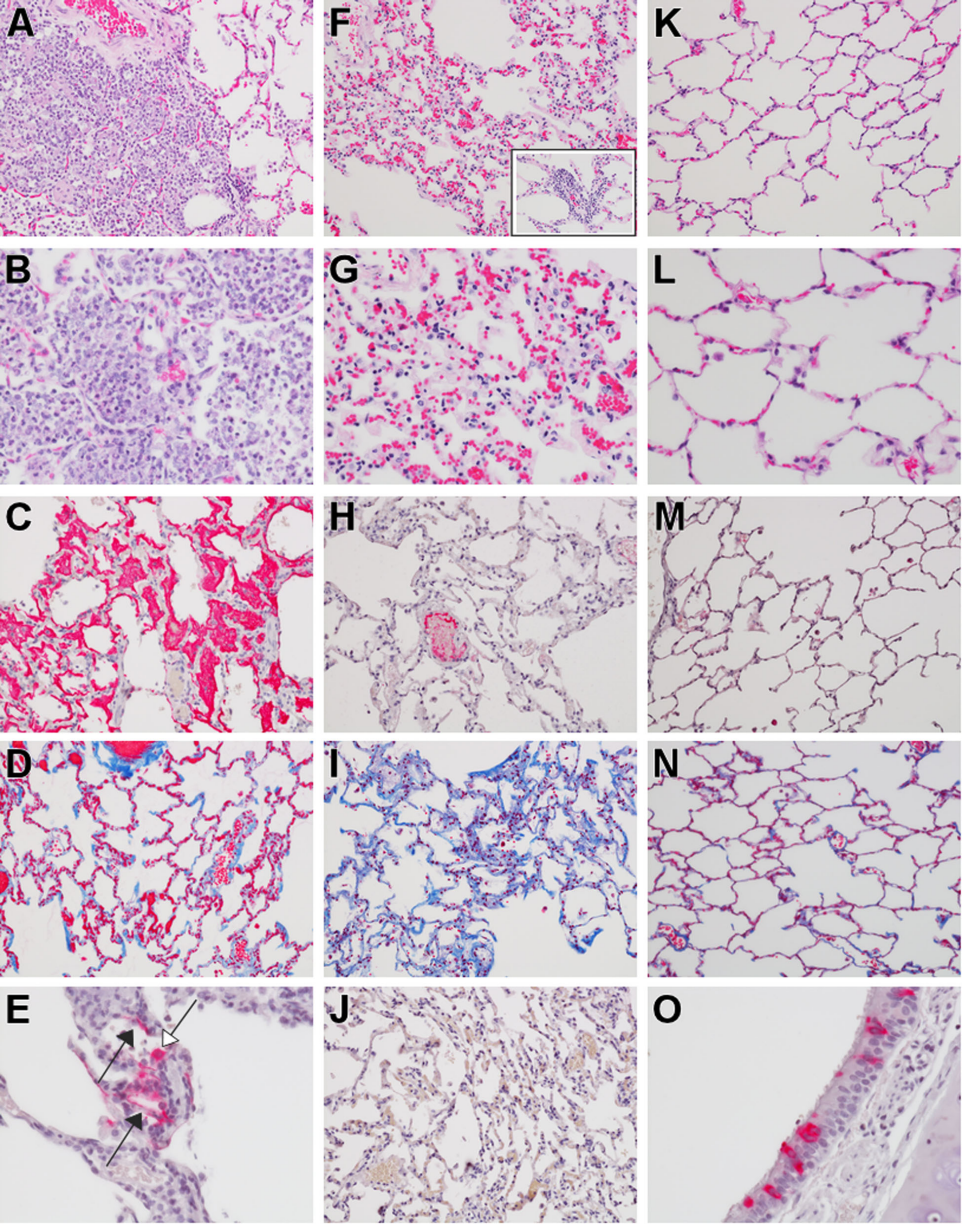
Comparative pulmonary histologic lesions in AGMs infected with SARS-CoV-2. Representative tissues of AGM from 5 dpi (**a**-**e** & **o**) and 34 dpi (**f**-**j**). SARS-CoV-2 naïve tissues from an AGM (**k**-**n**). H&E staining at low magnification (20x) (**a**, **f**, & **k**) and higher magnification (40x) (**b**, **g**, **l**, and **f** inset) of pulmonary alveolar septae and alveolar spaces. Moderate neutrophilic bronchiolitis and alveolitis and mild interstitial pneumonia with congestion (**a** & **b**) Moderate lymphohistocytic interstitial pneumonia with congestion, mild alveolar wall fibrosis (**f** & **g**) and moderate perivascular lymphocytic cuffs (**f** inset). No significant lesions (**k** & **l**). IHC for anti-fibrin antigen (red) (**c**, **h** & **m**). Alveolar spaces are partially to completely flooded with fibrin (**c**) Minimal intravascular fibrin labeling (**h**) and no significant fibrin immunolabeling (**m**). Trichrome special stain for collagen (blue) (**d**, **i** & **n**). Minimal to mild alveolar wall collagen deposition (**d**) moderate alveolar wall collagen deposition (**i**) and minimal collagen staining of alveolar wall basement membranes (**n**). IHC labeling for anti-SARS-CoV2 antigen (red) (**e**, **j** & **o**). IHC positive type I pneumoncytes (black arrows) and type II pneumoncytes (white arrow) localized with alveolar inflammation (**e**), No immonolabeling (**j**) and IHC positive labeling of respiratory epithelium of the bronchus (**o**). Images captured at 20x (**m**, **d**, **i**, **n**, & **j**) and 40x (**c**, **h**, **e**, & **o**)

Histologically, all three AGMs euthanized at 5 dpi developed mild multifocal neutrophilic bronchointerstitial pneumonia (Fig. 5a-e, o). Histologic features include acute inflammation centered within the airways of terminal bronchioles with occasional flooding of adjacent alveolar spaces with neutrophils, macrophages, fibrin, edema, hemorrhage, mucous and rarely multinucleated giant cells **(5A, B)**. In lesser-affected regions alveolar septate were expanded with mixed inflammatory cells and alveolar spaces contain increased numbers of alveolar macrophages with scattered red blood cells. Ulcerative tracheobronchitis was also present in all three AGMs and characterized by multifocal epithelial erosion associated with underlying hemorrhage, fibrin accumulation and infiltrating acute inflammation. Polymerized fibrin, highlighted by IHC, colocalized with acute inflammation within the bronchial lumen, alveolar spaces, alveolar walls and ulcerated regions of the trachea and bronchus (Fig. 5c). Fibrin was also present within medium and small caliber vessels but was not associated with an obvious adherent thrombus. Trichrome stain of representative lung sections identified modest collagen deposition within multifocal regions of alveolar septae (Fig. 5d). IHC for SARS-CoV-2 antigen was positive in all three AGMs associated with pulmonary lesions. Positive IHC labeling was noted diffusely within the cytoplasm of respiratory epithelium of the bronchus (Fig. 5o) and less in type I and type II pneumocytes (Fig. 5e).

Histologically, all three AGMs euthanized at 34 dpi developed moderate multifocal chronic interstitial pneumonia Fig. 5f-j). Histologic features include expansion of alveolar septae with macrophages, lymphocytes, and very rarely neutrophils (Fig. 5f, g). Wispy, pale eosinophilic, acellular material also multifocally expanded the alveolar walls and stained as immature collagen with trichrome staining (Fig. 5i). Polymerized fibrin was present within medium and small caliber vessels but was not associated with an obvious adherent thrombus (Fig. 5h). No immunolabeling for SARS-CoV-2 was noted with IHC in any of the examined tissue sections from this 34 dpi cohort (Fig. 5j).

## Discussion

We previously reported the development of the AGM as a promising animal model of human COVID-19 [10]. Other studies have subsequently reported similar findings [11, 12]. The focus of the current study was to assess a more natural route of human exposure, specifically an exposure mimicking an infection resulting from mucosal exposure to infectious droplets expelled from close quarter exposure to a sneeze, cough, or even speech in order to begin characterization of lung pathology in the early convalescence phase of COVID-19. The disease resulting from the i.n. MAD challenge was largely reflective of that observed with the combination of the i.t. and i.n. routes except it appeared to be somewhat milder in terms of length of any fever, less severe signs of pneumonia as evidenced by reduced alveolar flooding, and a lower prevalence of SARS-CoV-2 infection [10]. However, the MAD-infected AGMs still developed virus-induced pneumonia and viral shedding was detected into the early convalescence period. While it appears that inclusion of direct i.t. instillation of SARS-CoV-2 as an exposure route may result in a more severe disease in AGMs, it is also possible that animal to animal variability may have contributed to the modest difference between the studies. SARS-CoV-2 infection of humans results in a wide spectrum of disease ranging from asymptomatic to severe and fatal disease so it is not unexpected that there could be variability among AGMs as well. While the current study employed female AGMs because of animal availability at the time the work was initiated, gender did not affect the outcome when compared to similar studies [10–12].

Coagulation dysfunction is a consistent observation in human COVID-19 and has been associated with disease severity [18–22]. Here, we performed a limited analysis of blood clotting times (PT and aPTT) and circulating fibrinogen levels to begin to characterize the coagulopathy in SARS-CoV-2-infected AGMs. Transient increases in aPTT and in circulating fibrinogen levels were observed during the acute phase of infection. Increases in PT and/or aPTT have been linked to severe human COVID-19 cases in some but not all studies [18–22]. However, nearly all severe COVID-19 cases have been associated with high levels of fibrinogen [20–22].

Our findings regarding lung injury in the three AGMs that were euthanized at 34 dpi during early convalescence are consistent with the limited human COVID-19 studies that have been reported so far. For example, a recent study of fifty-seven COVID-19 patients in China was completed during the early convalescence phase, approximately 30 days after discharge [23]. The study included 40 non-severe cases and 17 severe cases. Thirty-one patients (54.3%) had abnormal CT findings while abnormalities were detected in the pulmonary function tests in 43 (75.4%) of the patients. In a second human study, 21 patients recovering from COVID-19 (without severe respiratory distress during the disease course), had lung abnormalities visible on chest CT at 10 days after initial onset of symptoms [24]. While other studies suggest that some of the abnormalities may be resolved over time [25, 26] more research needs to be conducted in this area.

Regarding histopathology, human data is particularly sparse. One small study performed thoracoscopies with blebs resection and pleurectomies on performed on the 16th and 23rd days from symptoms onset of two patients [27]. Despite well-known pulmonary damages induced during the acute phase of COVID-19, the late-phase gross and histological changes include nonspecific chronic reparative lesions, similarly to what we have described in the AGMs at 34 dpi. Grossly in the human study, there was non-specific diffuse pulmonary congestion, edema and hemorrhagic necrosis. Histologically, the main lesions were focused on alveolar damage with mildly thickened alveolar interstitial tissues with fibrosis and mononuclear cellular infiltration (lymphocytes, plasma cells and multinucleate giant cells). Intravascular hemorrhagic thrombosis was also noted in these specimens.

In summary, we have expanded on our previous development of the combined i.n. and i.t. inoculation model of SARS-CoV-2 in AGMs. Importantly, while AGMs challenged with SARS-CoV-2 via the LMA MAD exhibited apparently milder clinical illness and disease, hallmark features from our previous study were still apparent, notably the development of viral pneumonia during the acute phase. The AGM COVID-19 model should be useful in future studies to assess disease and develop interventions that improve recovery.

## Supplementary information

**Additional file 1: Figure S1**: Longitudinal temperature monitoring of SARS-CoV-2 infected AGMs. Temperature was longitudinally monitored for all animals via surgically-implanted temperature loggers (see Materials and methods). Data shown for all animals begins 1 day prior to infection (− 1) and terminates in the morning of day 5 post-infection, when AGM-1, AGM-2, and AGM-3 were euthanized. Periods of elevated temperature are indicated in red. Arrows denote time of challenge.

**Additional file 2: Table S1**: Clinical description and outcome of African green monkeys following SARS-CoV-2 challenge. Days after SARS-CoV-2 challenge are in parentheses. All reported findings are in comparison to baseline (d0) values. Decreased appetite is defined as some food but not all food consumed from the previous day. Anorexia is defined as no food consumed from the previous day. Lymphocytopenia, monocytopenia, erythrocytopenia, thrombocytopenia, neutropenia, eosinopenia, and basopenia are defined by a ≥ 35% drop in numbers of lymphocytes, monocytes, erythrocytes, platelets, neutrophils, eosinophils, and basophils, respectively. Lymphocytosis, monocytosis, neutrophilia, eosinophilia, and basophilia are defined by a 100% or greater increase in numbers of lymphocytes, monocytes, neutrophils, eosinophils, or basophils, respectively. Hyperglycemia is defined as a 100% or greater increase in levels of glucose. Hypoglycemia is defined by a ≥ 25% decrease in levels of glucose. Hypoalbuminemia is defined by a ≥ 25% decrease in levels of albumin. Hypoproteinemia is defined by a ≥ 25% decrease in levels of total protein. Hypoamylasemia is defined by a ≥ 25% decrease in levels of serum amylase. Hypocalcemia is defined by a ≥ 25% decrease in levels of serum calcium. Hypercapnia was defined as having a partial CO2 > 4 mmHg over d0 baseline values. (ALT) alanine aminotransferase, (AST) aspartate aminotransferase, (ALP) alkaline phosphatase, (CRE) Creatinine, (CRP) C-reactive protein, (Hct) hematocrit, (Hgb) hemoglobin.

**Additional file 3: Table S2**: Gross lung lesion severity scores in AGMs infected with SARS-CoV-2

## Data Availability

The data supporting the conclusions of this article are included within the article.

## Abbreviations

aPTT: Activated partial thromboplastin time
AGM: African green monkey
BAL: Bronchoalveolar lavage
COVID-19: Coronavirus Disease 2019
IHC: Immunohistochemistry
i.n.: Intranasal
i.t.: Intratracheal
MAD: Mucosal Atomization Device
PFU: Plaque forming unit
PT: Prothrombin time
SARS-CoV-2: Severe acute respiratory syndrome coronavirus 2
